# 3,3-Dimethyl-1,1-(propane-1,3-di­yl)diimidazol-1-ium tetra­bromido­cadmate(II)

**DOI:** 10.1107/S1600536810031211

**Published:** 2010-08-11

**Authors:** Ling-hua Zhuang, Chun-ling Zheng, Chang-sheng Wang, Ai-lin Yuan, Guo-wei Wang

**Affiliations:** a Department of Applied Chemistry, College of Science, Nanjing University of Technology, Nanjing 210009, People’s Republic of China; b Department of Light Chemical Engineering, College of Food Science and Light Industry, Nanjing University of Technology, Nanjing 210009, People’s Republic of China

## Abstract

The title compound, (C_11_H_18_N_4_)[CdBr_4_], was prepared by an anion exchange. The dihedral angle between the two planar imidazolium rings in the cation is 74.4 (4)°. The crystal packing is stabilized by weak inter­molecular C—H⋯Br hydrogen bonds between the cation and the tetrahedral anion, building up a three-dimensionnal network.

## Related literature

For the properties and applications of ionic liquids, see: Welton (1999 ▶); Nicholas *et al.* (2004 ▶); Yu *et al.* (2007 ▶). For dicationic ionic liquids, see: Jared *et al.* (2005 ▶); Liang *et al.* (2008 ▶); Song *et al.* (2009 ▶); Geng *et al.* (2010 ▶). For related structures, see: Jared *et al.* (2005 ▶); Liang *et al.* (2008 ▶). For bond-length data, see: Allen *et al.* (1987 ▶).
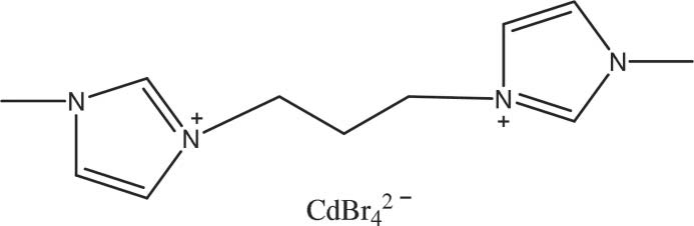


## Experimental

### 

#### Crystal data

(C_11_H_18_N_4_)[CdBr_4_]
*M* *_r_* = 638.33
Monoclinic, 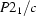

*a* = 8.5050 (17) Å
*b* = 15.876 (3) Å
*c* = 13.836 (3) Å
β = 96.07 (3)°
*V* = 1857.7 (6) Å^3^
*Z* = 4
Mo *K*α radiation
μ = 9.78 mm^−1^
*T* = 293 K
0.20 × 0.10 × 0.10 mm

#### Data collection

Enraf–Nonius CAD-4 diffractometer
Absorption correction: ψ scan (North *et al.*, 1968 ▶) *T*_min_ = 0.245, *T*_max_ = 0.442
3383 measured reflections
3383 independent reflections
1874 reflections with *I* > 2σ(*I*)
3 standard reflections every 200 reflections  intensity decay: 1%

#### Refinement

*R*[*F* ^2^ > 2σ(*F* ^2^)] = 0.062
*wR*(*F* ^2^) = 0.144
*S* = 0.95
3383 reflections
183 parameters
H-atom parameters constrained
Δρ_max_ = 0.72 e Å^−3^
Δρ_min_ = −0.77 e Å^−3^

### 

Data collection: *CAD-4 Software* (Enraf–Nonius, 1989 ▶); cell refinement: *CAD-4 Software*; data reduction: *XCAD4* (Harms & Wocadlo, 1995 ▶); program(s) used to solve structure: *SHELXS97* (Sheldrick, 2008 ▶); program(s) used to refine structure: *SHELXL97* (Sheldrick, 2008 ▶); molecular graphics: *ORTEPIII* (Burnett & Johnson, 1996 ▶), *ORTEP-3 for Windows* (Farrugia, 1997 ▶) and *PLATON* (Spek, 2009 ▶); software used to prepare material for publication: *SHELXL97*.

## Supplementary Material

Crystal structure: contains datablocks global, I. DOI: 10.1107/S1600536810031211/dn2593sup1.cif

Structure factors: contains datablocks I. DOI: 10.1107/S1600536810031211/dn2593Isup2.hkl

Additional supplementary materials:  crystallographic information; 3D view; checkCIF report

## Figures and Tables

**Table 1:** Hydrogen-bond geometry (Å, °)

*D*—H⋯*A*	*D*—H	H⋯*A*	*D*⋯*A*	*D*—H⋯*A*
C7—H7*A*⋯Br2	0.97	2.67	3.515 (15)	145
C1—H1*C*⋯Br3^i^	0.96	2.93	3.819 (13)	155
C4—H4*A*⋯Br3^ii^	0.93	2.70	3.606 (11)	164
C5—H5*B*⋯Br3^iii^	0.97	2.84	3.765 (11)	161
C8—H8*A*⋯Br4^iv^	0.93	2.86	3.699 (12)	151

Symmetry codes: (i) 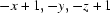; (ii) 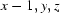; (iii) 
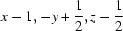
; (iv) 
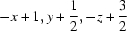
.

**Fig. 1.**
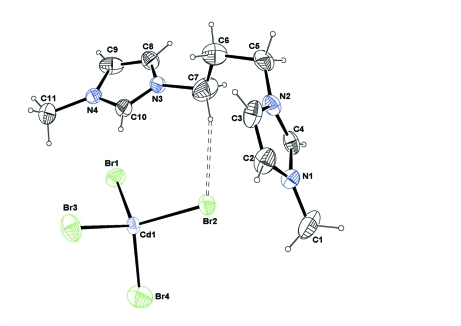
A view of the molecular structure of (I) showing the atom-numbering scheme. Displacement ellipsoids are drawn at the 30% probability level. Hydrogen are represented as small spheres of arbitrary radii. Hydrogen bond is shown as dashed line.

**Fig. 2.**
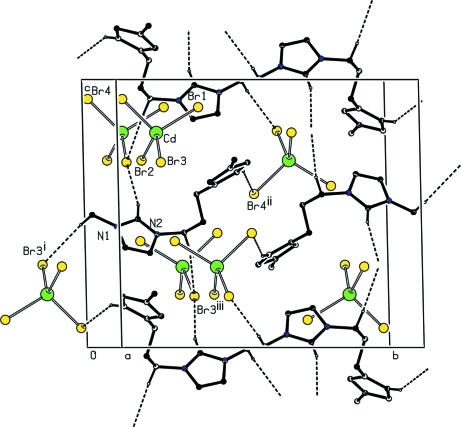
Partial packing view showing the C-H···Br Hydrogen bonding pattern. Hydrogen bonds are drawn as dashed lines. H atoms not involved in hydrogen bonding have been omitted for clarity. [Symmetry codes : (i) -x+1, -y, -z+1; (ii) -x+1, y+1/2, -z+3/2; (iii) x-1, -y+1/2, z-1/2.

## supplementary crystallographic information

### Comment

Ionic liquids (ILs) are generally formed by an organic cation and a weakly coordinating anion. They have enjoyed considerable research interests in recent years because of their unique properties such as high thermal stability, non-volatility, non-flammability, high ionic conductivity, wide electrochemical window and miscibility with organic compounds (Welton, 1999; Nicholas *et al.*, 2004; Yu *et al.*, 2007). Geminal dicationic ionic liquids have been shown to possess superior physical properties in terms of thermal stability and volatility compared to traditional ionic liquids (ILs) (Jared *et al.*, 2005; Liang *et al.*, 2008; Song *et al.*, 2009). As part of our ongoing studies on new Geminal dicationic ionic liquids (Geng *et al.*, 2010), we report here the crystal structure of the title compound (I).

In (I) (Fig. 1), the bond lengths and angles are within normal ranges (Allen *et al.*, 1987). The two imidazolium rings are of course planar but make a dihedral angle of 74.4 (4)° which is much larger than the values reported for related compound as C_11_H_18_N_4._Br_2_, 21.5° (Jared *et al.*, 2005) or C_11_H_18_N_4._2PF_6_ ,6.1 (2)°(Liang *et al.*, 2008). The dication of the title structure has a highly twisted conformation with the two imidazolium rings almost perpendicular to the C_3_ plane (angles of C5C6C7-N2C4N1C2C3 and C5C6C7-C8C9N4C10N3 are 79.4 (8)° and 86.1 (6)°, respectively), which are significantly lower than those observed in C_11_H_18_N_4._Br_2_ (106.8 (7)° and 92.6 (6)°, respectively) (Jared *et al.*, 2005).

Weak intermolecular C—H···Br hydrogen bonds between tetrabromide cadmium anions and imidazolium cations build up a three dimensionnal network. (Table 1, Fig.2).

### Experimental

A solution of 1,3-dibromide propane(10.1 g, 0.05 mol) in methanol(30 ml) was slowly added to a solution of 1-methylimidazole(9.4 g, 0.11 mol) in methanol(30 ml) at room temperature. The reaction mixture was then refluxed for 6 h. After evaporation of the solvent, the residue was washed with diethyl ether and dichloromethane, then dried in vacuum to obtain ionic liquid 3,3-dimethyl-1,1-(propane-1,3-diyl)- diimidazol-1-ium dibromide (a white solid ionic liquid).

A solution of above mentioned dibromide ionic liquid (3.66 g, 0.01 mol) in methanol(20 ml) was slowly added to a methanol solution of cadmium dibromide (3.44 g, 0.02 mol). The reaction mixture was stirred at room temperature for 3 h. After evaporation of the solvent, the residue was washed with methanol, then dried in vacuum to obtain title compound (I), 3,3-dimethyl-1,1-(propane-1,3-diyl)- diimidazol-1-ium tetrabromide cadmium(II)(yield 84%). *M*.p. 452–454 K.

Crystals of (I) suitable for X-ray diffraction were obtained by slow evaporation of methanol. ^1^H NMR (DMSO, δ, p.p.m.) 8.82 (s, 2 H), 7.49 (d, 4 H), 4.37 (t, 4 H), 3.93 (s,6 H), 2.57 (t, 4 H).

### Refinement

All H atoms attached to C atoms fixed geometrically and treated as riding with C—H = 0.96 Å (methyl), 0.97 Å (methylene) or 0.93 Å (aromatic) with U_iso_(H) = 1.2U_eq_(C) or U_iso_(H) = 1.5U_eq_(Cmethyl).

### Crystal data

**Table:**

(C_11_H_18_N_4_)[CdBr_4_]	*F*(000) = 1200
*M**_r_* = 638.33	*D*_x_ = 2.282 Mg m^−^^3^
Monoclinic, *P*2_1_/*c*	Mo *K*α radiation, λ = 0.71073 Å
Hall symbol: -P 2ybc	Cell parameters from 25 reflections
*a* = 8.5050 (17) Å	θ = 9–13°
*b* = 15.876 (3) Å	µ = 9.78 mm^−^^1^
*c* = 13.836 (3) Å	*T* = 293 K
β = 96.07 (3)°	Block, white
*V* = 1857.7 (6) Å^3^	0.20 × 0.10 × 0.10 mm
*Z* = 4	

### Data collection

**Table:**

Enraf–Nonius CAD-4 diffractometer	1874 reflections with *I* > 2σ(*I*)
Radiation source: fine-focus sealed tube	*R*_int_ = 0.000
graphite	θ_max_ = 25.3°, θ_min_ = 2.0°
ω/2θ scans	*h* = −10→10
Absorption correction: ψ scan (North *et al.*, 1968)	*k* = 0→19
*T*_min_ = 0.245, *T*_max_ = 0.442	*l* = 0→16
3383 measured reflections	3 standard reflections every 200 reflections
3383 independent reflections	intensity decay: 1%

### Refinement

**Table:**

Refinement on *F*^2^	Primary atom site location: structure-invariant direct methods
Least-squares matrix: full	Secondary atom site location: difference Fourier map
*R*[*F*^2^ > 2σ(*F*^2^)] = 0.062	Hydrogen site location: inferred from neighbouring sites
*wR*(*F*^2^) = 0.144	H-atom parameters constrained
*S* = 0.95	*w* = 1/[σ^2^(*F*_o_^2^) + (0.066*P*)^2^] where *P* = (*F*_o_^2^ + 2*F*_c_^2^)/3
3383 reflections	(Δ/σ)_max_ < 0.001
183 parameters	Δρ_max_ = 0.72 e Å^−^^3^
0 restraints	Δρ_min_ = −0.77 e Å^−^^3^

### Special details

**Table:**

Geometry. All esds (except the esd in the dihedral angle between two l.s. planes) are estimated using the full covariance matrix. The cell esds are taken into account individually in the estimation of esds in distances, angles and torsion angles; correlations between esds in cell parameters are only used when they are defined by crystal symmetry. An approximate (isotropic) treatment of cell esds is used for estimating esds involving l.s. planes.
Refinement. Refinement of *F*^2^ against ALL reflections. The weighted *R*-factor *wR* and goodness of fit *S* are based on *F*^2^, conventional *R*-factors *R* are based on *F*, with *F* set to zero for negative *F*^2^. The threshold expression of *F*^2^ > σ(*F*^2^) is used only for calculating *R*-factors(gt) *etc*. and is not relevant to the choice of reflections for refinement. *R*-factors based on *F*^2^ are statistically about twice as large as those based on *F*, and *R*- factors based on ALL data will be even larger.

### Fractional atomic coordinates and isotropic or equivalent isotropic displacement parameters (Å^2^)

**Table:**

	*x*	*y*	*z*	*U*_iso_*/*U*_eq_	
N1	0.2634 (10)	0.0761 (6)	0.4518 (6)	0.045 (2)	
N2	0.2576 (10)	0.2095 (6)	0.4221 (7)	0.052 (2)	
N3	0.5789 (10)	0.3667 (6)	0.6079 (7)	0.060 (3)	
N4	0.8099 (10)	0.3936 (6)	0.6694 (6)	0.047 (2)	
C1	0.2360 (16)	−0.0015 (9)	0.4950 (10)	0.095 (5)	
H1A	0.2116	0.0075	0.5604	0.142*	
H1B	0.3289	−0.0360	0.4959	0.142*	
H1C	0.1488	−0.0293	0.4584	0.142*	
C2	0.3518 (14)	0.0926 (9)	0.3730 (9)	0.066 (4)	
H2A	0.4038	0.0519	0.3400	0.079*	
C3	0.3505 (13)	0.1739 (8)	0.3527 (9)	0.064 (4)	
H3A	0.3989	0.2014	0.3045	0.076*	
C4	0.2128 (11)	0.1516 (8)	0.4794 (8)	0.050 (3)	
H4A	0.1545	0.1609	0.5315	0.060*	
C5	0.2341 (14)	0.3012 (8)	0.4287 (9)	0.068 (4)	
H5A	0.1515	0.3121	0.4704	0.081*	
H5B	0.1983	0.3228	0.3646	0.081*	
C6	0.3818 (15)	0.3485 (8)	0.4682 (9)	0.074 (4)	
H6A	0.4681	0.3349	0.4304	0.089*	
H6B	0.3630	0.4087	0.4637	0.089*	
C7	0.4240 (18)	0.3240 (9)	0.5717 (11)	0.096 (5)	
H7A	0.4352	0.2634	0.5770	0.115*	
H7B	0.3414	0.3416	0.6106	0.115*	
C8	0.5739 (14)	0.4410 (8)	0.6537 (8)	0.057 (3)	
H8A	0.4833	0.4729	0.6583	0.068*	
C9	0.7154 (16)	0.4612 (8)	0.6906 (9)	0.067 (4)	
H9A	0.7468	0.5103	0.7240	0.080*	
C10	0.7192 (13)	0.3354 (8)	0.6172 (8)	0.055 (3)	
H10A	0.7515	0.2842	0.5934	0.065*	
C11	0.9894 (14)	0.3870 (10)	0.7007 (12)	0.107 (6)	
H11A	1.0261	0.4384	0.7320	0.160*	
H11B	1.0442	0.3778	0.6444	0.160*	
H11C	1.0094	0.3408	0.7450	0.160*	
Cd1	0.77770 (8)	0.15445 (5)	0.80145 (6)	0.0423 (2)	
Br1	0.79089 (15)	0.29343 (8)	0.89683 (10)	0.0652 (4)	
Br2	0.50899 (14)	0.13451 (8)	0.69780 (10)	0.0643 (4)	
Br3	1.00029 (15)	0.14239 (11)	0.69122 (10)	0.0832 (5)	
Br4	0.8109 (2)	0.04053 (11)	0.92709 (13)	0.1013 (6)	

### Atomic displacement parameters (Å^2^)

**Table:**

	*U*^11^	*U*^22^	*U*^33^	*U*^12^	*U*^13^	*U*^23^
N1	0.048 (6)	0.046 (6)	0.043 (5)	−0.003 (5)	0.011 (4)	−0.003 (4)
N2	0.040 (6)	0.056 (6)	0.061 (6)	0.011 (5)	0.004 (5)	−0.009 (5)
N3	0.046 (6)	0.034 (6)	0.092 (8)	−0.013 (4)	−0.031 (5)	0.002 (5)
N4	0.044 (5)	0.057 (6)	0.042 (5)	−0.002 (5)	0.010 (4)	0.021 (5)
C1	0.097 (11)	0.091 (11)	0.094 (11)	−0.056 (9)	0.004 (9)	0.006 (10)
C2	0.062 (9)	0.070 (9)	0.070 (9)	−0.008 (7)	0.029 (7)	−0.016 (7)
C3	0.054 (8)	0.077 (10)	0.063 (8)	−0.019 (7)	0.020 (6)	−0.018 (7)
C4	0.031 (6)	0.081 (9)	0.037 (6)	0.006 (6)	0.005 (5)	−0.009 (7)
C5	0.064 (9)	0.077 (10)	0.055 (8)	−0.001 (7)	−0.027 (6)	0.004 (7)
C6	0.094 (10)	0.073 (9)	0.056 (8)	0.005 (8)	0.009 (7)	0.000 (8)
C7	0.122 (14)	0.065 (10)	0.099 (12)	−0.021 (9)	0.005 (10)	−0.013 (8)
C8	0.054 (8)	0.062 (9)	0.052 (7)	0.002 (6)	−0.001 (6)	−0.003 (6)
C9	0.103 (11)	0.043 (7)	0.052 (7)	0.010 (8)	0.004 (7)	−0.009 (6)
C10	0.045 (7)	0.061 (8)	0.057 (7)	−0.006 (6)	0.001 (6)	0.015 (6)
C11	0.053 (9)	0.080 (11)	0.183 (18)	−0.017 (8)	−0.009 (10)	0.034 (11)
Cd1	0.0313 (4)	0.0491 (5)	0.0475 (5)	−0.0006 (4)	0.0080 (3)	−0.0051 (4)
Br1	0.0724 (9)	0.0536 (8)	0.0706 (9)	−0.0055 (6)	0.0119 (7)	−0.0081 (6)
Br2	0.0457 (7)	0.0684 (9)	0.0760 (9)	−0.0047 (6)	−0.0061 (6)	−0.0025 (7)
Br3	0.0583 (8)	0.1260 (14)	0.0684 (9)	0.0112 (9)	0.0214 (7)	−0.0020 (9)
Br4	0.1088 (13)	0.0956 (12)	0.1011 (12)	0.0178 (10)	0.0180 (10)	0.0049 (10)

### Geometric parameters (Å, °)

**Table:**

N1—C4	1.342 (13)	C5—C6	1.514 (16)
N1—C1	1.400 (15)	C5—H5A	0.9700
N1—C2	1.412 (13)	C5—H5B	0.9700
N2—C4	1.297 (13)	C6—C7	1.490 (17)
N2—C3	1.423 (14)	C6—H6A	0.9700
N2—C5	1.475 (15)	C6—H6B	0.9700
N3—C10	1.286 (13)	C7—H7A	0.9700
N3—C8	1.341 (14)	C7—H7B	0.9700
N3—C7	1.519 (15)	C8—C9	1.297 (15)
N4—C10	1.362 (13)	C8—H8A	0.9300
N4—C9	1.391 (13)	C9—H9A	0.9300
N4—C11	1.545 (14)	C10—H10A	0.9300
C1—H1A	0.9600	C11—H11A	0.9600
C1—H1B	0.9600	C11—H11B	0.9600
C1—H1C	0.9600	C11—H11C	0.9600
C2—C3	1.321 (17)	Cd1—Br4	2.5036 (19)
C2—H2A	0.9300	Cd1—Br3	2.5608 (16)
C3—H3A	0.9300	Cd1—Br1	2.5673 (15)
C4—H4A	0.9300	Cd1—Br2	2.5858 (16)
			
C4—N1—C1	126.2 (10)	C7—C6—C5	108.9 (11)
C4—N1—C2	105.5 (9)	C7—C6—H6A	109.9
C1—N1—C2	128.3 (11)	C5—C6—H6A	109.9
C4—N2—C3	110.5 (10)	C7—C6—H6B	109.9
C4—N2—C5	127.7 (11)	C5—C6—H6B	109.9
C3—N2—C5	121.5 (11)	H6A—C6—H6B	108.3
C10—N3—C8	111.6 (9)	C6—C7—N3	108.2 (11)
C10—N3—C7	128.6 (11)	C6—C7—H7A	110.0
C8—N3—C7	118.6 (11)	N3—C7—H7A	110.0
C10—N4—C9	109.1 (10)	C6—C7—H7B	110.0
C10—N4—C11	126.2 (11)	N3—C7—H7B	110.0
C9—N4—C11	124.7 (12)	H7A—C7—H7B	108.4
N1—C1—H1A	109.5	C9—C8—N3	109.2 (11)
N1—C1—H1B	109.5	C9—C8—H8A	125.4
H1A—C1—H1B	109.5	N3—C8—H8A	125.4
N1—C1—H1C	109.5	C8—C9—N4	105.0 (11)
H1A—C1—H1C	109.5	C8—C9—H9A	127.5
H1B—C1—H1C	109.5	N4—C9—H9A	127.5
C3—C2—N1	110.6 (11)	N3—C10—N4	105.0 (10)
C3—C2—H2A	124.7	N3—C10—H10A	127.5
N1—C2—H2A	124.7	N4—C10—H10A	127.5
C2—C3—N2	103.7 (11)	N4—C11—H11A	109.5
C2—C3—H3A	128.1	N4—C11—H11B	109.5
N2—C3—H3A	128.1	H11A—C11—H11B	109.5
N2—C4—N1	109.6 (9)	N4—C11—H11C	109.5
N2—C4—H4A	125.2	H11A—C11—H11C	109.5
N1—C4—H4A	125.2	H11B—C11—H11C	109.5
N2—C5—C6	113.6 (10)	Br4—Cd1—Br3	108.87 (6)
N2—C5—H5A	108.9	Br4—Cd1—Br1	105.57 (6)
C6—C5—H5A	108.9	Br3—Cd1—Br1	112.06 (6)
N2—C5—H5B	108.9	Br4—Cd1—Br2	108.95 (6)
C6—C5—H5B	108.9	Br3—Cd1—Br2	109.04 (6)
H5A—C5—H5B	107.7	Br1—Cd1—Br2	112.23 (5)

### Hydrogen-bond geometry (Å, °)

**Table:**

*D*—H···*A*	*D*—H	H···*A*	*D*···*A*	*D*—H···*A*
C7—H7A···Br2	0.97	2.67	3.515 (15)	145.
C1—H1C···Br3^i^	0.96	2.93	3.819 (13)	155.
C4—H4A···Br3^ii^	0.93	2.70	3.606 (11)	164.
C5—H5B···Br3^iii^	0.97	2.84	3.765 (11)	161.
C8—H8A···Br4^iv^	0.93	2.86	3.699 (12)	151.

Symmetry codes: (i) −*x*+1, −*y*, −*z*+1; (ii) *x*−1, *y*, *z*; (iii) *x*−1, −*y*+1/2, *z*−1/2; (iv) −*x*+1, *y*+1/2, −*z*+3/2.
